# Impact of congenital heart disease on outcomes among pediatric patients hospitalized for influenza infection

**DOI:** 10.1186/s12887-020-02344-x

**Published:** 2020-09-28

**Authors:** Laxmi V. Ghimire, Fu-Sheng Chou, Anita J. Moon-Grady

**Affiliations:** 1 Section of Pediatrics and Section of Cardiology, Department of Medicine, Lakes Region General Hospital, Laconia, NH USA; 2 Department of Pediatrics, Loma Linda University, Loma Linda, CA USA; 3 Clinical Pediatrics, Division of Pediatric Cardiology, Department of Pediatrics, University of California, San Francisco, 550 16th Street 5th Floor, San Francisco, CA 94158 USA

**Keywords:** Influenza, Congenital heart disease, Kids’ inpatient database, Hospitalization, Pediatrics

## Abstract

**Background:**

Young children and those with chronic medical conditions are at risk for complications of influenza including cardiopulmonary compromise. Here we aim to examine risks of mortality, clinical complications in children with congenital heart disease (CHD) hospitalized for influenza.

**Methods:**

We analyzed data from in-hospital pediatric patients from 2003, 2006, 2009, 2012 and 2016 using the nationally representative Kids Inpatient Database (KID). We included children 1 year and older and used weighted data to compare the incidence of in-hospital mortality and rates of complications such as respiratory failure, acute kidney injury, need for mechanical ventilation, arrhythmias and myocarditis.

**Results:**

Data from the KID estimated 125,470 children who were admitted with a diagnosis of influenza infection. Out of those, 2174(1.73%) patients had discharge diagnosis of CHD. Children with CHD who required hospitalization for influenza had higher in-hospital mortality (2.0% vs 0.5%), with an adjusted OR (aOR) of 2.8 (95% CI: 1.7–4.5). Additionally, acute respiratory failure and acute kidney failure were more likely among patients with CHD, with aOR of 1.8 (95% CI: 1.5–2.2) and aOR of 2.2 (95% CI: 1.5–3.1), respectively. Similarly, the rate of mechanical ventilatory support was higher in patients with CHD compared to those without, 14.1% vs 5.6%, aOR of 1.9 (95% CI: 1.6–2.3). Median length of hospital stay in children with CHD was longer than those without CHD [4 (IQR: 2–8) days vs. 2 (IQR: 2–4) days]. Outcomes were similar between those with severe vs non-severe CHD.

**Conclusions:**

Children with CHD who require hospital admission for influenza are at significantly increased risk for in-hospital mortality, morbidities, emphasizing the need to reinforce preventative measures (e.g. vaccination, personal hygiene) in this particularly vulnerable population.

## Background

Influenza infection generally presents as an acute self-limiting illness in healthy children, but it can cause significant morbidity and mortality in high-risk children. The Center for Disease Control and Prevention (CDC) estimated around 500,000 individuals were hospitalized, causing more than 34,000 deaths during the 2018–2019 influenza season [1]. Children <5 years, and <2 years and those with chronic medical conditions are at risk of complications from influenza, including pneumonia and acute respiratory failure.

Congenital heart disease (CHD) is prevalent in 1% of the population [2]. These children are at increased risks for complications of influenza and therefore routine annual influenza immunization is strongly recommended in these children. However, there are only a handful of studies in the literature reporting mortality and complications related to influenza among children with chronic diseases [3, 4]. Similarly, there is also very limited information on complications of influenza infection among children with CHD. Here, we report a recent nationwide cross-sectional retrospective study based on the Kids’ Inpatient Database (KID) of the United States to examine risks of mortality, in-hospital complications including respiratory failure, acute kidney injury and need for mechanical ventilation, as well as length of hospital stay as a result of influenza infection among children with CHD.

## Methods

### Study population and variables

We analyzed data of hospital discharge records of patients with the nationally representative Kids’ Inpatient Database (KID) for 2003, 2006, 2009, 2012 and 2016. The data was compiled by the Agency for Healthcare Research and Quality (AHRQ) and was generated for Healthcare Cost and Utilization Project (HCUP) in collaboration with public and private statewide data organizations. The KID is a stratified, cross-sectional database that includes discharge data for approximately 10% of newborn discharges and 80% of other discharges in the United States. KID is published every 3–4 years and the latest data available is from 2016.

Using International Classification of Disease, Ninth and Tenth Revision, Clinical Modification (ICD-9-CM and ICD-10-CM), we identified hospitalized children with CHD, influenza infection, and other variables studied. To include only CHD, we excluded patent ductus arteriosus, single umbilical artery and other anomalies of peripheral vascular system from analysis. Please see Supplementary Table 1 for ICD-9-CM and ICD-10-CM codes used in the manuscript. We included children 1 year and older, excluding the infants(< 1 year) to minimize the confounding effect on the outcome variables, as most of the surgeries for CHD occur during infancy and we also wanted to minimize the effect of patent ductus arteriosus especially on preterm infants, which is minimal after infancy.

### Outcome variables

The primary outcome of interest was comparison of in-hospital mortality between those children with influenza infection with and without concomitant CHD. Secondary outcomes were acute respiratory failure, acute kidney injury, need for invasive mechanical ventilation (IMV), non-invasive mechanical ventilation (NIMV), myocarditis, tachyarrhythmias, heart block, sudden cardiac arrest, need for ECMO and length of hospital stay.

### Statistical analysis

We performed descriptive and inferential statistics using the KID complex survey design, taking into account for clusters, strata, and weighting. For continuous variables such as age and length of stay, we reported median with interquartile range (IQR). Weight-adjusted Chi-square tests were used for categorical variables and weight-adjusted Wilcoxon signed rank tests for continuous variables as the continuous variables were not evenly distributed.

The variables used in the multivariable analysis were carefully selected after rigorous review of the literature. During selection, we were careful to identify variables that had ICD codes that were reliable and consistent. For regression modeling, univariable analyses of each variable of interest were performed first, followed by a multivariable analysis incorporating additional variables (age, sex, race/ethnicity, discharge quarter, year of admission, history of asthma, presence of respiratory and musculoskeletal congenital anomalies, presence of chromosomal anomalies) to determine the effects of covariates and confounding variables on the outcome of interest. We performed logistic regression analysis for the odds ratios (ORs) of the risk of mortality, acute respiratory failure, acute kidney injury and need for mechanical ventilation with and without concomitant CHD. We assessed differences in length of hospital stay by using multiple linear regression analysis.

We then performed analysis to compare the risk between severe and non-severe CHD. Severe CHD includes truncus arteriosus, d-transposition of great arteries(d-TGA), double outlet right ventricle(DORV), l-transposition of great arteries(L-TGA), tetralogy of Fallot, hypoplastic left heart syndrome(HLHS), other single ventricles, atrio-ventricular septal defect(AVSD), pulmonary atresia, tricuspid atresia, interrupted aortic arch, total anomalous pulmonary venous return(TAPVR). Every congenital heart disease without ICD codes for severe CHD were included as non-severe CHD. In addition, if a child has a diagnosis of severe CHD and non-severe CHD, they are counted as severe CHD, e.g. if a child has d-transposition of great arteries(d-TGA) and VSD, they are counted as d-TGA and not as VSD.

Weights provided by HCUP were used in all analyses to account for the complex sampling design and clustering for the analysis. All statistical analyses were performed using Stata statistical software (version 15.1), R version 3.6.0 [5] and R Studio 1.2 [6]. (http://www.R-project.org) Complex survey design of the KID was accounted for using the *survey* package [7]. Figures were produced using the *ggplot2* and *patchwork* packages. Tables were generated using the *tableone* package [8].

## Results

**Fig. 1:**
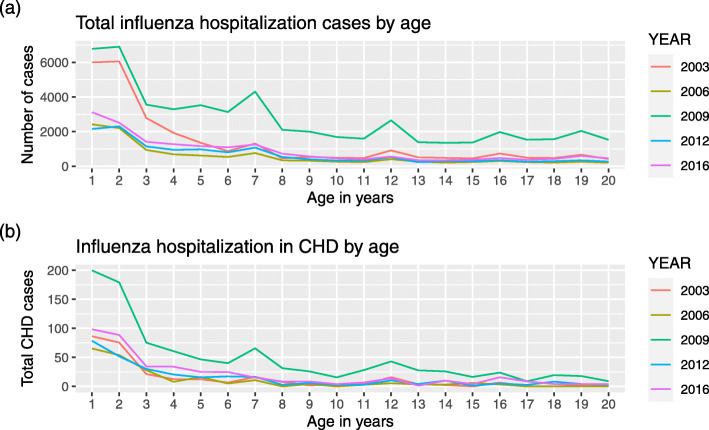
**a**. Total influenza hospitalization cases by age. **b**. Children with CHD who are hospitalized by Influenza infection by age

The retrieved records based on the inclusion and exclusion criteria estimated 125,470 children who were admitted with a diagnosis of influenza. Of those, 2174 (1.73%) had a diagnosis of CHD. 57,055 (45.5%) were female. Median age of children with influenza was 5 (IQR: 2–11) years. The number of cases were higher in 2009 compared to other years probably due to the H1N1 influenza pandemic during that year. More children were hospitalized in the October to December quarter compared to the other quarters. Hospitalization was more common in younger children compared to the older children and adolescents and this finding was consistent overall all years studied (Fig. 1).

Regarding the chronic medical conditions in the hospitalized children, history of asthma was present in 27.8% of hospitalized children, while only 0.64% of the children had congenital anomalies of the respiratory system. 1.9% of the children had chromosomal anomalies.

The overall in-hospital mortality rate for children hospitalized with influenza was 0.54% (*n* = 687). 5.4% of children had respiratory failure and 4.6% required invasive mechanical ventilation. Median hospitalization length was 2(IQR: 2–4) days. Baseline characteristics of children with influenza are reported in Table 1.

**Table 1:** Characteristics of pediatric patients hospitalized for influenza infection

Variables	Cases, n, (%)
Total influenza cases	125,470
Age, years (median)	5 (IQR: 2–11)
Female	57,055 (45.7%)
Race	
White	49,405 (47.7%)
Black	20,821 (20.1%)
Hispanic	23,371 (22.6%)
Others	9907 (9.6%)
Year	
2003	27,503 (21.9%)
2006	11,702 (9.3%)
2009	54,487 (43.4%)
2012	13,849 (11.0%)
2016	17,929 (14.3%)
Discharge quarter	
Jan-Mar	36,903 (29.4%)
Apr-Jun	15,719 (12.5%)
Jul-Sep	10,232 (8.2%)
Oct-Dec	62,542 (49.9%)
CHD^b^	2174 (1.73%)
Severe CHD	613 (0.5%)
Non-severe CHD	1561 (1.2%)
Comorbid conditions	
Asthma	34,931 (27.8%)
Congenital respiratory anomalies	815 (0.7%)
Congenital musculoskeletal anomalies	1326 (1.1%)
Chromosomal anomalies	2362 (1.9%)
Complications	
Respiratory failure	6714 (5.4%)
Acute kidney injury	1534 (1.2%)
Invasive mechanical ventilation (IMV)	5791 (4.6%)
Non-invasive mechanical ventilation (NIMV)	1943 (1.5%)
Myocarditis	199 (0.2%)
Tachyarrhythmias	514 (0.5%)
Heart block/conduction disorders	312 (0.3%)
Sudden cardiac arrest	250 (0.2%)
ECMO^a^	211 (0.2%)
In-hospital mortality	687 (0.54%)

^a^Extracorporeal membrane oxygenation

^b^*CHD* Congenital heart disease

The children admitted with influenza who have concomitant CHD were younger than those without CHD, median age 3 (IQR: 2–7) years vs 5 (IQR: 2–11) years, *P* < 0.001.

History of asthma was more common in children without CHD [27.9% (*n* = 34,410) in non-CHD patients vs 24.0% (*n* = 521) in CHD patients], while congenital anomalies, specifically respiratory anomalies, musculoskeletal anomalies and chromosomal anomalies, were significantly more common in CHD group (Table 2).

**Table 2:** Comparison of characteristics and in-hospital complications between those with and without CHD

Variables	Presence of CHD	
	Yes	No
Total influenza cases	2174	123,296
Age, years (median, IQR)	3 (2–7)	5 (2–11)*
Female	977 (44.9%)	56,078 (45.8%)
Race		
White	858 (45.0%)	48,547 (47.8%)
Black	298 (15.6%)	20,522 (20.2%)
Hispanic	575 (30.2%)	22,796 (22.4%)
Others	174 (9.1%)	9733 (9.6%)
Discharge quarter		
Jan-Mar	678 (31.3%)	36,224 (29.4%)
Apr-Jun	398 (18.3%)	15,321 (12.4%)
Jul-Sep	193 (8.9%)	10,038 (8.2%)
Oct-Dec	900 (41.5%)	61,642 (50.0%)
Comorbid conditions		
Asthma	521 (24.0%)	34,410 (27.9%)*
Congenital respiratory anomalies	112 (5.1%)	704 (0.6%)*
Congenital musculoskeletal anomalies	98 (4.5%)	1228 (1.0%)*
Chromosomal anomalies	426 (19.6%)	1936 (1.6%)*
Complications		
Respiratory failure	267 (12.3%)	6447 (5.2%)*
Acute kidney injury	60 (2.8%)	1474 (1.2%)*
Invasive mechanical ventilation (IMV)	262 (12.0%)	5529 (4.5%)*
Non-invasive mechanical ventilation (NIMV)	67 (3.1%)	1877 (1.5%)*
Myocarditis	< 11 (NA)^a^	193 (0.2%)
Tachyarrhythmias	54 (2.5%)	460 (0.4%)*
Heart block/conduction disorders	75 (3.4%)	238 (0.2%)*
Sudden cardiac arrest	18 (0.8%)	232 (0.2%)*
ECMO^a^	12 (0.6%)	199 (0.2%)*
In-hospital mortality	44 (2.0%)	643 (0.5%)*

^a^Extracorporeal membrane oxygenation

CHD: Congenital heart disease

^*^P-value is < 0.001 for these comparisons

^a^Healthcare Cost and Utilization Project (HCUP) restricts subjects < 11 to be reported

Regarding in hospital complications between those with CHD and without CHD, tachyarrhythmias [2.5% (*n* = 54) vs 0.4% (*n* = 460), *p* < 0.001] conduction disorders [3.4% (*n* = 75) vs 0.2% (*n* = 238), p < 0.001] and sudden cardiac arrest [0.8% (*n* = 18) vs 0.2% (*n* = 232), *p* < 0.001] were more common in the CHD group. (Table 2).

**Fig. 2:**
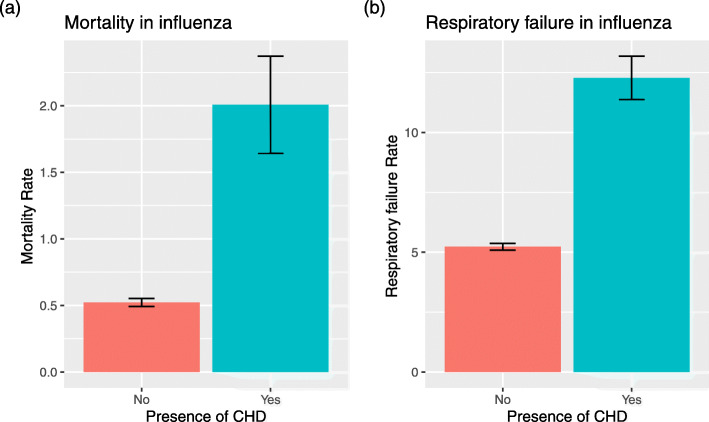
**a**. In-hospital mortality rate between those with CHD vs those without CHD. **b** Rate of acute respiratory failure in those with CHD vs those without, amongst children hospitalized by influenza infection. The differences in mortality and rate of respiratory failure was statistically significant between children hospitalized with influenza with CHD vs those without CHD. Multivariable logistic regression test was used to calculate statistical significance

In-hospital mortality was higher in children with CHD 2.0% (*n* = 44) compared to those without CHD 0.52%(*n* = 643). (Fig. 2a.) In univariable logistic regression analysis, patients with CHD had increased risk of mortality by 3.9 fold, with an OR of 3.9 (95% CI 2.7–5.7, *p* < 0.001). We then performed multivariable logistic regression analysis with additional confounders including age, sex, year of admission, race, discharge quarter, the presence of chronic medical conditions specifically asthma, respiratory and chronomosomal anomalies. In this model, we found that patients with CHD had an increased odds of mortality by 2.8 fold, with an adjusted odds ratio (aOR) of 2.8 (95% CI: 1.7–4.5, *p* < 0.001). In this model, the presence of respiratory and chromosomal congenital anomalies increased risk of mortality (Table 3).

**Table 3:** Predictions of mortality in children hospitalized with influenza, on a multivariable logistic regression model

Variables	Odds ratio with 95% CI	*P*-value
CHD	2.8 (1.7–4.5)	< 0.001
Age	1.1 (1.07–1.1)	< 0.001
Sex (Female)	0.98 (0.8–1.2)	0.85
Race		
White	Reference	–
Black	0.96 (0.7–1.3)	0.79
Hispanic	1.4 (1.1–1.8)	0.02
Others	1.4 (1.0–2.0)	0.03
Year		
2003	Reference	–
2006	1.2 (0.7–2.1)	0.42
2009	0.92 (0.6–1.3)	0.66
2012	0.69 (0.4–1.1)	0.091
2016	0.7 (0.45–1.1)	0.14
Discharge quarter		
Jan-Mar	Reference	–
Apr-Jun	1.6 (1.2–2.2)	0.002
Jul-Sep	1.8 (1.3–2.6)	< 0.001
Oct-Dec	0.96 (0.7–1.3)	0.77
Comorbid conditions		
Asthma	0.37 (0.3–0.5)	< 0.001
Congenital respiratory anomalies	2.2 (1.1–4.6)	0.03
Congenital musculoskeletal anomalies	3.0 (1.8–5.1)	< 0.001
Chromosomal anomalies	2.9 (1.9–4.6)	< 0.001

Similarly, we performed univariable and multivariable regression analyses to compare the risk of acute respiratory failure and acute kidney injury in patients with and without CHD. In a multivariable logistic regression model, acute respiratory failure was more common among those with CHD compared to those without CHD, with an aOR of 1.8 (95% CI: 1.5–2.2, *p* < 0.001) (Fig. 2b, Table 4). In this model, history of asthma, respiratory and chromosomal congenital anomalies increased risk of mortality significantly. The risk of acute kidney injury was also higher in patients with CHD even after adjusting for confounders, with an aOR of 2.2 (95% CI: 1.5–3.1, *p* < 0.001) (Supplementary Table 2). The rate of mechanical ventilatory support was higher in patients with CHD compared to those without [14.1% vs 5.6%, aOR of 1.9 (95% CI: 1.6–2.3, p < 0.001)] (Supplementary Table 3).

**Table 4:** Predictions of respiratory failure in children hospitalized with influenza, on a multivariable logistic regression model

Variables	Odds ratio with 95% CI	*P*-value
CHD	1.85 (1.5–2.2)	< 0.001
Age	1.03 (1.03–1.04)	< 0.001
Sex (Female)	0.95 (0.9–1.1)	0.16
Race		
White	Reference	–
Black	0.91 (0.8–1.0)	0.07
Hispanic	1.0 (0.9–1.1)	0.67
Others	1.2 (1.1–1.4)	0.005
Year		
2003	Reference	–
2006	1.6 (1.2–2.2)	0.003
2009	1.8 (1.4–2.3)	< 0.001
2012	1.9 (1.5–2.5)	< 0.001
2016	3.4 (2.6–4.5)	< 0.001
Discharge quarter		
Jan-Mar	Reference	–
Apr-Jun	1.5 (1.3–1.7)	< 0.001
Jul-Sep	1.5 (1.3–1.7)	< 0.001
Oct-Dec	1.2 (1.1–1.2)	0.005
Comorbid conditions		
Asthma	1.1 (1.1–1.2)	0.002
Congenital respiratory anomalies	2.5 (1.9–3.4)	< 0.001
Congenital musculoskeletal anomalies	2.8 (2.3–3.4)	< 0.001
Chromosomal anomalies	2.2 (1.9–2.6)	< 0.001

Median length of hospital stay in children with CHD was longer than those without CHD [4 (IQR: 2–8) days vs. 2 (IQR: 2–4) days, p < 0.001].

We then divided the CHD group into those with severe CHD and non-severe CHD and performed analysis to compare the incidence of complications between these two groups. We found that most of the major complications were very similar between these groups. (Supplementary Table 4).

## Discussion

Using the KID 2003, 2006, 2009, 2012 and 2016, a large national database of more than 125,000 pediatric influenza-associated hospitalizations, we reported the following major findings: 1) presence of CHD increased the risk of in-hospital mortality in children with influenza infection, 2) concomitant CHD was more likely to be associated with acute respiratory failure, acute kidney injury, need for invasive and noninvasive mechanical ventilation, myocarditis and need for ECMO, 3) length of hospital stay was higher in CHD with influenza, and 4) there were no significant differences in primary and secondary outcomes between severe and non-severe CHD.

Higher rates of in-hospital all cause mortality in patients with CHD seem reasonable since children with CHD could already have limited cardiac and respiratory reserves and therefore may not tolerate increased cardiorespiratory demand during active infections. Additionally, children who have had thoracotomy could have restrictive lung physiology making them more prone to pulmonary complications compared to otherwise healthy children. Specifically, in those children with CHD, introduction of pro-inflammatory and oxidative mediators by an acute systemic inflammatory response caused by an influenza infection may lead to further comprise in cardiac function [9]. In addition, we also found that the respiratory and cardiac complications are higher in those patients with CHD. These complications may also contribute to overall increased mortality.

Another important finding we report is the similarities in the mortality and major hospital complications between those with severe CHD and non-severe CHD. Most of the severe CHD patients would have undergone surgical repair in infancy, and after surgical repair of the lesions, their cardiac hemodynamics probably improved. That potentially is the reason that they are just as vulnerable as those non-severe CHD, but not significantly higher.

The cardiovascular complications of influenza have been reported, including myocarditis, arrhythmias, cardiac arrest and death [10]. However, the exact mechanism how influenza infection causes adverse cardiovascular outcomes remains elusive. There is some evidence suggesting that activation of the inflammatory pathways may be responsible for acute myocardial injury and dysfunction [11].

We found that the prevalence of asthma was more common in children without CHD compared to those hospitalized for influenza with CHD (27.9% vs 24%). We are not entirely sure if this will hold true for children who are not hospitalized. Literature lacks large studies reporting association between asthma and CHD.

The World Health Organization estimated 5–10% of adults and 20–30% of children are infected with influenza worldwide, resulting in 290,000–650,000 deaths annually [12]. We also know that in the pediatric population, influenza vaccination offers significant protection against severe influenza disease and influenza-related hospitalization [13, 14]. Therefore, annual influenza vaccination for children is recommended to reduce adverse health impacts and to prevent severe complications. However, vaccination coverage for children in the influenza season 2018–2019 was only 62.6% according to the Center for Disease Control and Prevention of the United States [15]. There are very limited data on the vaccination rate for influenza among children with heart diseases including CHD. A recent Israeli study reported 36% vaccination rate among children with heart disease [16]. In a report on influenza-related death of 291 pediatric patients from the 2010–2011 to the 2013–2014 seasons, 30 children had CHD or acquired cardiac disease, only 23% of whom received influenza vaccination [17]. Studies have reported that influenza vaccination has been associated with a reduced risk for all-cause mortality in patients with heart failure or with other cardiovascular disease, emphasizing the importance of influenza vaccination, especially in those with chronic medical conditions including CHD patients [18, 19].

Using the KID, our study provided a larger sample size based on population sampling of hospitalized pediatric patients with influenza, which was the main strength of our study. However, there were multiple limitations with this approach. First, data in the KID were not collected for research, but were for medical coding and billing. Therefore, incorrect or missing information may potentially exist. Second, KID captures only inpatient records, so the findings may not be applicable to the outpatient settings and to those children with chronic cardiac disease who are not hospitalized. Third, there was no information on the accuracy of the diagnosis. This data does not include information on prior influenza vaccination and how influenza cases were confirmed and verified. Moreover, we were not able to study the influence of vaccination status on clinical outcomes using this database. In addition, due to limitation of the database, we could not report details on anatomy and physiology of CHD like the status of residual lesion, whether they have decreased ventricular function, functional status of children and timing of surgical repair or palliation.

## Conclusions

In conclusion, our findings indicate that children with influenza infection and a concomitant CHD are at increased risk of in-hospital mortality and adverse clinical outcomes. Vaccination rates in children, especially those with chronic disease conditions such as CHD are lower, despite the evidence that influenza vaccination decreases severity of the illness, mortality and complications in patients with chronic medical conditions. We recommend health care providers, families, and official organizations to work together to increase influenza vaccination rates in all children, especially in those with chronic medical conditions, to prevent morbidity and mortality.

## Supplementary information

**Additional file 1: Supplementary Table 1.** Variables and corresponding ICD-9-CM and ICD-10-CM codes. **Supplementary Table 2.** Predictions of acute kidney injury in children hospitalized with influenza, on a multivariable logistic regression model. **Supplementary Table 3.** Predictions of mechanical ventilation in children hospitalized with influenza, on a multivariable logistic regression model. **Supplementary Table 4.** Characteristics and complication comparing severe vs non-severe CHD children hospitalized for influenza.

## Data Availability

Because of limitations of the KID data use agreement and availability of the data directly from the Agency for Healthcare Research and Quality, the data, analytic methods, and study materials will not be made available to other researchers for purposes of reproducing the results or replicating the procedure.

## Abbreviations

CHD: Congenital heart disease
ECMO: Extracorporeal membrane oxygenation
AHRQ: Agency for Healthcare Research and Quality (AHRQ)
HCUP: Healthcare Cost and Utilization Project (HCUP)
KID: Kids inpatient database
