# Supplementary material for: Impact of congenital heart disease on outcomes among pediatric patients hospitalized for influenza infection
Source: BMC Pediatr. 2020 Sep 28;20:450. doi: 10.1186/s12887-020-02344-x (PMC7520971; doi:10.1186/s12887-020-02344-x)
Supplement: Supplementary file 1 — Additional file 1: Supplementary Table 1. Variables and corresponding ICD-9-CM and ICD-10-CM codes. Supplementary Table 2. Predictions of acute kidney injury in children hospitalized with influenza, on a multivariable logistic regression model. Supplementary Table 3. Predictions of mechanical ventilation in children hospitalized with influenza, on a multivariable logistic regression model. Supplementary Table 4. Characteristics and complication comparing severe vs non-severe CHD children hospitalized for influenza. [file 12887_2020_2344_MOESM1_ESM.docx]

**Supplementary tables:**

Supplementary Table 1: Variables and corresponding ICD-9-CM and ICD-10-CM codes

| Variables | ICD-9-CM code | ICD-10-CM code |
| --- | --- | --- |
| Influenza infection | 488.xx, 487.0, 487.1,487.8 | J10.xx, J11.xx, J09X, J09X1, J09X2, J09X3, J09X9 |
| Acute respiratory failure | 518.81, 518.83,518.84,799.1 | J96.0, J96.0x, J96.9, J96.9x |
| Acute kidney injury | 584.5, 584.6, 584.7, 584.8, 584.9 | N17, N17.0, N17.1, N17.2, N17.8, N17.9 |
| Congenital heart disease(CHD) | 745.xx to 747.xx  Excluded:  PDA: 747.0  747.5 Single umbilical artery  747.6 Other anomalies of peripheral vascular system | Q20.xx to Q26.xx  Excluded:  PDA: Q25.0 |
| Invasive mechanical ventilation(IMV) | 93.01, 96.03, 96.04, 96.05, 96.70, 96.71, 96.72 | 0DH57BZ,0DH58BZ, 0BH17EZ, 0BH18EZ, 0B717DZ, 0B718DZ, 0BH07DZ, 0WHQ7YZ, 5A1935Z, 5A1945Z, 5A1955Z |
| Non-invasive mechanical ventilation(NIMV) | 93.90, 93.91 | 5A09357, 5A09457, 5A09557, 5A09358, 5A09458, 5A09558 |
| Asthma | 493.xx | J45.xx |
| Tachyarrhythmias | 427.0, 427.1, 427.41, 427.42, 427.31, 427.32 | I47.1, I47.2, I49.01, I49.0, I48.0, I48.3, I48.4 |
| Heart block | 426.12, 426.13, 426.0, 746.86, 426.6 | I44.1, I44.2 |
| Sudden cardiac arrest | 427.5 | I46.9 |
| ECMO* | 39.65 | 5A1522F, 5A1522G, 5A1522H |
| Congenital respiratory anomalies | 748.xx | Q30.xx-Q34.xx |
| Congenital musculoskeletal anomalies | 754.xx-756.xx | Q65.xx-Q79.xx |
| Chromosomal anomalies | 758.xx | Q90.xx-Q99.xx |

*Extracorporeal membrane oxygenation

Supplementary Table 2. Predictions of acute kidney injury in children hospitalized with influenza, on a multivariable logistic regression model

| Variables | Odds ratio with 95% CI | P-value |
| --- | --- | --- |
| CHD | 2.2 (1.5-3.1) | <0.001 |
| Age | 1.1 (1.1-1.2) | <0.001 |
| Sex(Female) | 0.8 (0.7-0.97) | 0.021 |
| Race | |  |
| White | Reference | - |
| Black | 0.98 (0.8-1.2) | 0.82 |
| Hispanic | 0.9 (0.7-1.1) | 0.23 |
| Others | 1.0 (0.8-1.3) | 0.9 |
| Year |  |  |
| 2003 | Reference | - |
| 2006 | 1.6 (0.9-2.9) | 0.10 |
| 2009 | 2.1 (1.4-3.1) | <0.001 |
| 2012 | 2.6 (1.7-4.0) | <0.001 |
| 2016 | 5.4 (3.5-8.3) | <0.001 |
| Discharge quarter | |  |
| Jan-Mar | Reference | - |
| Apr-Jun | 1.7 (1.4-2.0) | <0.001 |
| Jul-Sep | 1.5 (1.2-2.0) | 0.001 |
| Oct-Dec | 1.1 (0.91.3) | 0.44 |
| Comorbid conditions | |  |
| Asthma | 0.42 (0.35-0.51) | <0.001 |
| Congenital respiratory anomalies | 0.41 (0.1-1.7) | 0.22 |
| Congenital musculoskeletal anomalies | 1.5 (0.9-2.6) | 0.12 |
| Chromosomal anomalies | 2.1 (1.5-3.1) | <0.001 |

Supplementary Table 3. Predictions of mechanical ventilation in children hospitalized with influenza, on a multivariable logistic regression model

| Variables | Odds ratio with 95% CI | P-value |
| --- | --- | --- |
| CHD | 1.9 (1.6-2.3) | <0.001 |
| Age | 1.03 (1.02-1.04) | <0.001 |
| Sex(Female) | 0.95 (0.9-1.1) | 0.13 |
| Race | |  |
| White | Reference | - |
| Black | 1.0 (0.9-1.1) | 0.70 |
| Hispanic | 1.1 (0.9-1.1) | 0.44 |
| Others | 1.3 (1.2-1.5) | <0.001 |
| Year |  |  |
| 2003 | Reference | - |
| 2006 | 1.5 (1.1-2.0) | 0.003 |
| 2009 | 1.4 (1.1-1.9) | 0.002 |
| 2012 | 1.4 (1.1-1.9) | 0.003 |
| 2016 | 1.8 (1.4-2.3) | <0.001 |
| Discharge quarter | |  |
| Jan-Mar | Reference | - |
| Apr-Jun | 1.7 (1.5-1.9) | <0.001 |
| Jul-Sep | 1.4 (1.2-1.6) | <0.001 |
| Oct-Dec | 1.1 (0.98-1.2) | 0.09 |
| Comorbid conditions | |  |
| Asthma | 0.91 (0.8-0.99) | 0.03 |
| Congenital respiratory anomalies | 3.3 (2.6-4.3) | <0.001 |
| Congenital musculoskeletal anomalies | 3.1 (2.5-3.7) | <0.001 |
| Chromosomal anomalies | 2.5 (2.1-2.9) | <0.001 |

Supplementary Table 4: Characteristics and complication comparing severe vs non-severe CHD children hospitalized for influenza

| Variables | Type of CHD | | P-values |
| --- | --- | --- | --- |
|  | Non-severe | Severe |  |
| Total influenza cases | 1560 | 613 |  |
| Age, years (median, IQR) | 3 (2-7) | 3 (2-8) | 0.57 |
| Female | 721 (46.2%) | 256 (41.7%) | 0.14 |
| Race | | | |
| White | 600 (44.5%) | 258 (46.3%) | 0.54 |
| Black | 220 (16.4%) | 78 (14.0%) |  |
| Hispanic | 398 (29.5%) | 177 (31.8%) |  |
| Others | 129 (9.6%) | 45 (8.0%) |  |
| Discharge quarter | | | |
| Jan-Mar | 485 (31.1%) | 193 (31.6%) | 0.83 |
| Apr-Jun | 281 (18.1%) | 117 (19.1%) |  |
| Jul-Sep | 145 (9.3%) | 48 (7.9%) |  |
| Oct-Dec | 647 (41.5%) | 253 (41.4%) |  |
| Comorbid conditions | | | |
| Asthma | 420 (26.9%) | 101 (16.5%) | <0.001 |
| Congenital respiratory anomalies | 91 (5.9%) | 20 (3.3%) | 0.04 |
| Congenital musculoskeletal anomalies | 83 (5.3%) | 15 (2.5%) | 0.01 |
| Chromosomal anomalies | 318 (20.4%) | 108 (17.7%) | 0.27 |
| Complications | | | |
| Respiratory failure | 209 (13.4%) | 58 (9.4%) | 0.03 |
| Acute kidney injury | 41 (2.6%) | 19 (3.1%) | 0.61 |
| Invasive mechanical ventilation(IMV) | 202 (12.9%) | 60 (9.8%) | 0.18 |
| Non-invasive mechanical ventilation(NIMV) | 58 (3.7%) | 9 (1.4%) | 0.03 |
| Myocarditis | <11 | <11 | NA |
| Tachyarrhythmias | 30 (1.9%) | 24 (3.9%) | 0.02 |
| Heart block/conduction disorders | 38 (2.5%) | 37 (6.0%) | 0.001 |
| Sudden cardiac arrest | 13 (0.9%) | 4 (0.7%) | NA |
| ECMO^#^ | 10 (0.7%) | 2 (0.3%) | NA |
| In-hospital mortality | 32 (2.1%) | 11 (1.8%) | 0.75 |

^#^Extracorporeal membrane oxygenation

CHD: Congenital heart disease
